# Mining the *Penicillium expansum* Genome for Virulence Genes: A Functional-Based Approach to Discover Novel Loci Mediating Blue Mold Decay of Apple Fruit

**DOI:** 10.3390/jof9111066

**Published:** 2023-11-01

**Authors:** Dianiris Luciano-Rosario, Hui Peng, Verneta L. Gaskins, Jorge M. Fonseca, Nancy P. Keller, Wayne M. Jurick

**Affiliations:** 1 Food Quality Laboratory, USDA-ARS, Beltsville, MD 20705, USA; verneta.gaskins@usda.gov (V.L.G.); jorge.fonseca@usda.gov (J.M.F.); 2 Everglades Research and Education Center, Horticultural Sciences Department, University of Florida, Belle Glade, FL 33430, USA; huipeng@ufl.edu; 3 Department of Medical Microbiology and Immunology, University of Wisconsin, Madison, WI 53706, USA; npkeller@wisc.edu; 4 Department of Plant Pathology, University of Wisconsin, Madison, WI 53706, USA

**Keywords:** *Penicillium expansum*, virulence, random T-DNA mutagenesis, postharvest diseases

## Abstract

Blue mold, a postharvest disease of pome fruits, is caused by the filamentous fungus *Penicillium expansum*. In addition to the economic losses caused by *P. expansum*, food safety can be compromised, as this pathogen is mycotoxigenic. In this study, forward and reverse genetic approaches were used to identify genes involved in blue mold infection in apple fruits. For this, we generated a random T-DNA insertional mutant library. A total of 448 transformants were generated and screened for the reduced decay phenotype on apples. Of these mutants, six (T-193, T-275, T-434, T-588, T-625, and T-711) were selected for continued studies and five unique genes were identified of interest. In addition, two deletion mutants (Δ*t-625* and Δ*t-588*) and a knockdown strain (t-434*^KD^*) were generated for three loci. Data show that the ∆*t-588* mutant phenocopied the T-DNA insertion mutant and had virulence penalties during apple fruit decay. We hypothesize that this locus encodes a glyoxalase due to bioinformatic predictions, thus contributing to reduced colony diameter when grown in methylglyoxal (MG). This work presents novel members of signaling networks and additional genetic factors that regulate fungal virulence in the blue mold fungus during apple fruit decay.

## 1. Introduction

*Penicillium expansum* is a necrotrophic ascomycete and the causal agent of blue mold disease, a postharvest rot of apples and pears [1]. This filamentous fungus infects apples through wounds created during harvesting, handling, or packing, and the symptoms are most prevalent after storage, as this species can grow prolifically at very low temperatures. The prevalence of blue mold causes significant economic losses in the apple industry. For example, in the United States, blue mold is estimated to cause USD 50–250-million-dollar losses annually [1]. *P. expansum* not only threatens the apple industry, but also poses food safety concerns, as it produces patulin, which is difficult to eliminate from contaminated food products due to not being degraded by pasteurization [2,3]. The presence of patulin in processed food products is regulated by the FDA, the European Union, and the United Nations World Health Organization, which impose slightly different threshold levels, ranging from 10 to 50 ppb. Blue mold disease management relies primarily on fungicide applications, resulting in limited effectiveness due to the development of fungicide-resistant strains [4].

Due to the challenges in blue mold disease management and patulin reduction strategies, previous research has largely focused on understanding patulin biosynthesis and decontamination. While the molecular biology of *P. expansum* regarding virulence has also been investigated, only a few genes have been identified as significant and potential targets for disease-management potential. Many studies have focused on conserved virulence factors studied across the ascomycota fungi (e.g., creA, veA, laeA), and others have relied on mining-omics datasets [5,6,7,8]. While these approaches are high yielding, a forward genetics approach has not been implemented to date in *Penicillium expansum.* Historically, forward genetics approaches have produced many new advances toward gene discovery in diverse biological processes, including understanding the genetic mechanisms of disease caused by fungal plant pathogens [9,10]. One approach towards identifying and discovering genes of interest is through a random mutagenesis approach. Previous research in molecular plant pathology has successfully led to the identification of important loci for pathogenesis and/or virulence in diverse species [9,11,12,13,14,15].

This is the first study to generate a *P. expansum* library of random T-DNA insertional mutants using Agrobacterium-mediated transformation (AMT). We screened 448 of these transformants in apples for virulence defects and identified six single-spore T-DNA mutants (T-193, T-275, T-588/T-711, T-434, and T-625) that showed reduced lesion diameter upon apple inoculation when compared to the wild-type strain. They exhibited little to no growth penalty in vitro and contained a single T-DNA insertion in the genome. After performing TAIL-PCR, we identified the interrupted locus for each transformant. We obtained deletion mutants for two of these loci (∆*t-625* and ∆*t-588*) and a knockdown strain (t-434*^KD^*). Strains ∆*t-625* and t-434*^KD^* did not phenocopy the T-DNA mutants upon apple inoculations in three different cultivars. Interestingly, ∆*t-588* showed reduced lesion diameter when compared to the control strain in Honeycrisp apples. None of these mutants showed reduced nor enhanced tolerance to Congo Red or Sorbitol at different concentrations. We hypothesize that the gene interrupted in T-588 likely functions as a glyoxalase, as ∆*t-588* shows reduced colony diameter when compared to the control strains upon exposure to methylglyoxal. Hence, a new dimension of the virulence picture has been added to the complex process of decay, in which it is crucial for the fungus to effectively detoxify and/or metabolize toxic byproducts of the primary metabolism. The T-DNA mutant library also provides a functional platform for the fungal genetics community that can be screened for a particular phenotype in vitro or in vivo to understand the genetic basis of any given biological question.

## 2. Materials and Methods

### 2.1. Fungal Propagation and Media

*Penicillium expansum* strain R19, which was preserved in the USDA-ARS Food Quality Lab (FQL), was used in this study. Fungal growth conditions in culture and inoculation of apple fruit (‘Golden Delicious’ and ‘Ginger Gold’) were conducted following the methods used in a previous study by [16]. For the single-gene-deletion mutant generation, auxotrophic strains TDL 9.1 and TWW 12.1 (Pe21 background) were utilized. The strains were cultivated for conidial production in potato dextrose agar (PDA) (Becton, Dickinson and Company, Franklin Lakes, NJ, USA) or glucose minimal media (GMM) (10 g/L glucose, 1× Nitrate Salts, 1× Trace Elements, pH 6.5) at 25 °C for 5 to 7 days. GMM was also prepared using ammonium salts for culturing the knockdown strain TDL 37.1.

### 2.2. AMT of Penicillium expansum R19

The *Agrobacterium tumefaciens*-mediated transformation (AMT) of *Penicillium expansum* R19 was conducted following the protocol described by [17], with minor modifications. The *Agrobacterium tumefaciens* strain EHA105 was used as a T-DNA acceptor, and the binary plasmid pPK2 carrying the hygromycin B resistance gene (Hph) (Figure S1) was used as a T-DNA donor [18]. The plasmid was transferred into EHA105 cells using the heat-shock method [19]. A single colony of *A. tumefacies* carrying the plasmid pPK2 was cultured in 5 mL of YEB medium (5 g/L beef extract, 1 g/L yeast extract, 5 g/L peptone, 5 g/L sucrose, 0.5 g/L MgCl_2_) with kanamycin (100 μg mL^−1^) and rifampicin (50 μg mL^−1^) at 28 °C on a rotary shaker at 200 rpm until the OD600 reached 0.6–0.8. Cells from 2 mL of the culture were collected by centrifuge at 4000× *g* for 5 min and resuspended with 500 μL of liquid-induction medium (IM, 0.6 mg mL^−1^ MgSO_4_·7 H_2_O, 0.3 mg mL^−1^ NaCl, 0.01 mg mL^−1^ CaCl_2_, 0.01 mg mL^−1^ FeSO_4_, 0.4 mg mL^−1^ NH_4_NO_3_, 0.5% glycerol, 40 mM MES, 0.5 μg mL^−1^ H_3_BO_3_, 0.5 μg mL^−1^ ZnSO_4_·7H_2_O, 0.5 μg mL^−1^ CuSO_4_·5H_2_O, 0.5 μg mL^−1^ MnSO_4_·H_2_O, 0.5 μg mL^−1^ Na_2_MoO_4_·2H_2_O, and 0.2 mg mL^−1^ glucose, 1 mM potassium phosphate buffer, pH 5.8). The supernatant was discarded after the sample was centrifuged at 4000× *g* for 5 min. The bacterial pellets were diluted to OD600 = 0.15 with the IM supplemented with 200 μM acetosyringone, and then they were grown at 28 °C for 8–12 h on a rotary shaker at 200 rpm until the OD600 reached 0.3. *P. expansum* spores were harvested from 10-day-old PDA plates and diluted to 10^6^ mL^−1^ with sterilized water. The fungal-spore suspension and the induced *A. tumefaciens* culture were gently mixed by pipette at a ratio of 1:1, and set at room temperature for 2 h without shaking. A total of 200 μL of mixture was spread onto the surface of a nitrocellulose filter (0.45 μm) placed on an IM agar plate (liquid IM plus 1.0 mg mL^−1^ glucose and 1.5% agar) supplemented with 200 μM acetosyringone. The plates were incubated at 25 °C for 60 h. After cocultivation, the membranes were transferred onto a selection medium (PDA) containing 300 ng mL^−1^ cefotaxime and 100 ng mL^−1^ hygromycin B. After 4–5 days, visible colonies of transformants were isolated and streaked onto fresh selection plates. Consequent single colonies were selected for further experiments.

### 2.3. Fruit Wounding and Fungal Inoculation

Apple fruits (‘Golden Delicious’, ‘Ginger Gold’, ‘Fuji’, and ‘Honeycrisp’) were obtained from either the Penn State Fruit Research and Extension Center (Biglersville, PA) or local organic produce vendors. When possible, organic fruits were used, and when that was not possible, fruit without postharvest fungicide treatments or preharvest sprays with postharvest efficacy were procured. Pathogen inoculation was carried out with blemish- and disease-free fruits at a similar size following the published [16] method with minor modifications. Fruits were thoroughly washed with tap water and dried with a paper towels before sanitization with a spray of 75% ethanol. Fruits were dried with tissues and then wounded using a cylindrical wounding tool (3 mm deep × 3 mm diameter). The wounds were inoculated with 10 μL of 10^4^ conidia/mL suspension of *P. expansum* immediately after wounding. Twenty minutes after inoculation, boxes were loaded with inoculated fruits, covered with lids, and stored at room temperature. The lesion expansion of blue mold decay was measured at 4, 7, and 10 days, or every day for 7 days postinoculation (DPI).

### 2.4. Standard Molecular Methods

#### TAIL-PCR

Genomic DNA of the candidate transformants was extracted from mycelia using the Sigma Extract-N-Amp™ Plant PCR Kit (Sigma-Aldrich, St. Louis, MO, USA) according to the manufacturer’s instructions. Thermal asymmetric interlaced PCR (TAIL-PCR) was applied to identify the locus of T-DNA insertion. Degenerated primers included AD1 (5′-wagtgnagwancanaga-3′) and AD2 (5′-ntcgastwtsgwgtt-3′). Based on the DNA sequence of the T-DNA, a total of six specific primers in nested positions close to the left or right border of the T-DNA were designed and synthesized. Specific primers for the left border were LB1 (5′-gtgcctaatgagtgagctaactccc-3′), LB2 (5′-ctcacattaattgcgttgcgctc-3′), and LB3 (5′-gagcaattcggcgttaattcagt-3′). Specific primers for the right border were RB1 (5′-tggcactggccgtcgttttacaac-3′), RB2 (5′-aacgtcgtgactgggaaaaccct-3′), and RB3 (5′-cccttcccaacagttgcgca-3′). For the first-round PCR, components in 10 μL of reaction volume included 0.05 unit of HS ExTaq (TaKaRa Bio, Mountain View, CA, USA), 2 mM dNTP mix, 1 ng of genome DNA, 1 ppM T-DNA-specific primer (LB1 or RB1), and 10 ppM degenerated primer mix (AD1 and AD4). TAIL-PCR was performed according to the protocol used by [20], with some modification. For the secondary TAIL-PCR, the 10 μL of the reaction mix included 1 μL of a 1:100 dilution of the primary PCR products, 1 ppM LB2 or RB2 primer, and the composition of other reagents was the same as that of the primary PCR reaction mix. For the third TAIL-PCR, the 10 μL of reaction mix included 1 μL of a 1:100 dilution of the secondary PCR products and 1 ppm LB3 or RB3 primer, and other components were the same as those in the primary PCR reaction. Products of the third-round PCR were separated on 1% agarose gel with ethidium bromide. Single, prominent bands were purified with the Qiagen^®^ DNA Gel Extraction Kit (Qiagen, Frederick, MD, USA), and consequent DNA fragments were sequenced using T-DNA-specific primer (LB3 or RB3).

### 2.5. Identification of T-DNA Flanking Insertion

Genomic DNA of the strain R19 (wild-type) and the T-DNA mutants were extracted from mycelia using the Qiagen DNeasy Plant Mini Kit (Qiagen, Frederick, MD, USA). The T-DNA-induced mutation was identified through thermal asymmetric interlaced PCR (TAIL-PCR) according to a published protocol [20]. A total of thirteen primers, including three T-DNA left-border ones (Ppk2-LB1, 2, and 3), three right-border ones (Ppk2-LB1, 2, and 3), and seven arbitrary-degenerate ones (AD1-8), were used in the TAIL-PCR. Three rounds of PCR were run on a Bio-Rad PCR machine (CFX96 Touch™ Real-Time PCR Detection System) with EX Taq DNA Polymerase (TaKaRa Bio, Mountain View, CA, USA) using the product of the previous PCR as a template for the next. A common arbitrary primer and nested T-DNA-specific primers were employed in the PCR in a consecutive manner. Candidate bands from the second or third PCR were purified with the QIAquick PCR Purification Kit (Qiagen, Frederick, MD, USA), and resulting DNA fragments were subcloned with the Invitrogen TOPO TA Cloning Kit (Invitrogen, Frederick, MD, USA). These were sequenced with an ABI 3730 DNA Sequencer within the Iowa State University DNA Facility (Ames, IA, USA).

### 2.6. Gene Expression Analysis

Total RNA was isolated from frozen tissues using the RNeasy Plant Mini Kit (Qiagen, Frederick, MD, USA) according to the manufacturer’s instructions. Genomic DNA contamination was removed with TURBO DNase (Invitrogen, Frederick, MD, USA) on the column following the manufacturer’s instruction. Purified RNA was quantified with a NanoDrop 1000 spectrophotometer (Thermo Scientific, Waltham, MA, USA). The first strand of cDNA was synthesized with quantified RNA using iScriptTM Transcription Supermix (Bio-RAD, Hercules, CA, USA) following the manual. The primer pairs, CACGGTCTTGCCACTTGCTCGT/TCGTTTCGAGTACGTCGCGCTG and ACGTCGTCCCCATCTACGAG/GCTCAGCGAGGATCTTCATC were used for the expression analysis of *PeHSP* and the housekeeping gene actin (Genbank accession number XM_016739901). Quantitative PCR was performed with the CFX96 Touch™ Real-Time PCR Detection System (Bio-RAD, Hercules, CA, USA) using GoTaq^®^ qPCR Master Mix (Promega, Durham, NC, USA) and the following program: 95 °C for 2 min, followed by 45 cycles of 95 °C for 5 s and 60 °C for 15 s. A melt–curve analysis was performed to check the specificities of the PCR products. Relative expression of a given locus was normalized to the expression of actin and shown in fold changes (lowest value = 1) using the cycle threshold (Ct) 2^−ΔΔCt^ method [21]. The significance of the expression difference in various tissues at different timepoints was determined by Student’s *t*-test (*p* = 0.05) (Microsoft Excel 2010). All PCR experiments were performed in triplicate.

### 2.7. Southern Blot Analysis

Genomic DNA of the strain R19 and T-DNA mutants were isolated from mycelia using the Qiagen DNeasy Plant Mini Kit (Qiagen, Frederick, MD, USA) and digested with restriction endonuclease *PAC I*, separated by gel electrophoresis with 1.3% TAE agarose gel, and blotted onto Amersham Hybond-N+ nylon membranes (GE Healthcare Life Sciences, Laurel, MD, USA). A DNA fragment of 283 bp, including a portion of Hph DNA and a portion of Trp terminator, was used as the DNA hybridization probe. This fragment was amplified from the pPK2 plasmid with the primer pair TTCGGGCGTACACAAATCGCCC/AGGCACTCTTTGCTGCTTGGACA. The probes were labeled and detected using the DIG High Prime DNA Labeling and Detection Starter Kit II (Roche, Branchburg, NJ, USA) following the manual. Chemiluminescent signals on the membrane were recorded by Kodak X-ray film (8 × 10 inches).

### 2.8. Gene Deletion Construct Design

To obtain constructs for generating deletion mutants, primers were designed as described by [22,23] to obtain three distinct flanks. One of these flanks corresponded to the selectable marker pyrG from *Aspergillus fumigatus*, and the other two flanks corresponded to 1.5 kb upstream and downstream of the open reading frame (ORF) for the desired gene of interest. We used PCR to obtain independent amplicon fragments with primer, as listed in Table S1. After gel purification, double-joint PCR was performed and used as a template DNA for obtaining the constructs by a final PCR reaction. Regarding the knockdown constructs, the same principle was implemented, except that the primers were designed to insert the *Aspergillus nidulans* promoter niad/niia region upstream of the gene-of-interest’s ORF.

### 2.9. Polyethylene Glycol-Mediated Protoplast Transformation

The transformation process was carried out as described by [23,24], with some modifications. To obtain protoplasts, 10^9^ spores of strain TDL 9.1 or TWW 12.1 were incubated in 250 mL liquid GMM supplemented with yeast extract (YE), (1 g/L) uridine (0.56 g/L), and uracil (1.26 g/L) for 13.5 h at 25 °C and 280 rpm. After this, the cultures were centrifuged in bottles at 11,500× *g* for 2 min. After decanting the supernatant, the pellet was washed with sterile dH_2_O and transferred to a 20 mL tube, which was centrifuged at 25,000× *g* for 1 min. The supernatant was discarded, and approximately 200 µL of the pellet was transferred to the protoplast solution containing 90 mg of lysing enzymes from *Trichoderma harzianum* (Sigma-Aldrich, St. Louis, MO, USA), 60 mg of yatalase (TakaraBio, Kusatsu, Shiga, Japan) and 30 mL of Osmotic Medium (1 M MgSO_4_, 10 mM sodium phosphate buffer, pH 5.8). The solution was incubated in an Erlenmeyer flask for 4 h at 100 rpm at 28 °C. Then, the protoplasts were transferred to a 50 mL conical tube, and 10 mL of trapping buffer (0.6 M sorbitol, 10 mM Tris-HCl, pH 7.5) were added on top. This solution was centrifuged for 15 min at 1300× *g*. The remaining interphase was collected in a 15 mL conical tube and gently washed by adding 10 mL of STC buffer (1.2 M sorbitol, 10 mM CaCl_2_, 10 mM Tris-HCl, pH 7.5). This solution was then centrifuged for 7 min at 7800× *g*. The supernatant was discarded, and the pellet was resuspended in 1 mL of STC, then transferred to a 1.5 mL tube, where it was centrifuged for 15 s in a tabletop centrifuge at room temperature. The supernatant was discarded, and the pellet was resuspended in 300 µL of STC buffer. After this, 100 µL of the protoplast suspension, 15 µL of the purified construct, and 85 µL of STC buffer were incubated on ice for 50 min. After this period, 1.25 mL of a 60% PEG solution (60% PEG 4000, 50 mM CaCl_2_, 50 mM Tris-HCl, pH 7.5) was added to the tubes and incubated at room temperature for 20 additional minutes. Finally, 5 mL of STC buffer was added to the mixture, which was used to plate the transformants using an overlay method over sorbitol minimal media (SMM). Once the transformants grew, colonies were transferred to individual GMM plates and screened using PCR and Southern Blot.

### 2.10. Stress Plate Assays

GMM was supplemented with Congo Red (Sigma-Aldrich, St. Louis, MO, USA) at a final concentration of 10 µg/mL, 25 µg/mL, 100 µg/mL, or 200 µg/mL, sorbitol at a final concentration of 1 M, 1.2 M, or 1.5 M, and/or methylglyoxal (Sigma-Aldrich, St. Louis, MO, USA) at a final concentration of 0.1%. The plates were point-inoculated with total 100 spores, and incubated at 25 °C for 5 days for the Congo Red and Sorbitol or 12 days for the methylglyoxal.

### 2.11. Data Analysis

Statistical significance was analyzed with the *t*-test at each time point or 2-way ANOVA method, as appropriate. Multiple comparison of values was done with the Tukey HSD test. (* *p* < 0.05, ** *p* < 0.01,*** *p* < 0.001).

## 3. Results

### 3.1. Identification of Penicillium expansum AMT Virulence Mutants

**Figure 1:**
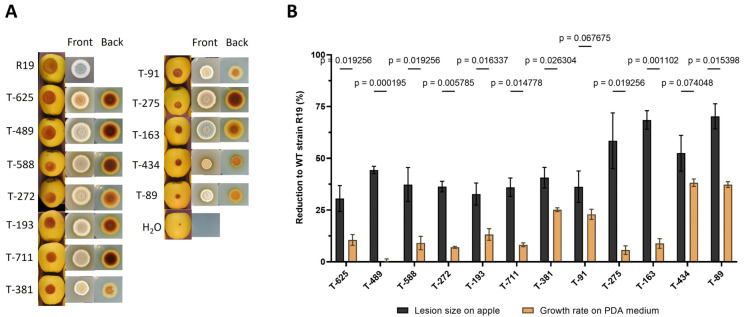
Morphology and virulence of T-DNA transformants. (**A**) Growth of T-DNA transformants on apples and PDA medium. Golden Delicious apples were inoculated with R19 or transformant spores and then stored in the dark at room temperature for 10 days. PDA plates were added R19 or transformant spores and then stored in an incubator at 25 °C for 6.5 days. (**B**) Virulence and growth reduction to wild-type strain R19 in transformants. The data represent the mean value (±SD) of three replicates, and comparisons were assessed using unpaired *t*-test with the Welch correction.

A collection of 448 transformants was generated through *Agrobacterium*-mediated transformation (AMT) using the T-DNA of pPK2*hph* as the insertional mutagen. All transformants were assessed for their impact on virulence in bioassays using three apples per transformant. Out of the 448 transformants, 50 putative mutants displayed a strong reduction (>20%) in virulence. To specifically isolate virulence-related genes using the candidate mutant pool, only those mutants that displayed virulence reduction on apples greater than a growth-rate change in culture medium were retested, again using three apples per transformant. In this way, 12 virulence mutants with a reproducible virulence defect were identified (Figure 1). Cultivation of these transformants on a rich medium revealed that T-DNA insertion has a specific impact on the morphology of fungi, such as the growth rate and color (Figure 1A). The colony of R19 has a blue ring with white edges. All transformants except for T163 showed various shades of brown pigmentation in media. Among the 12 mutants, T489 had the lowest growth-rate change in the culture medium but displayed 44.4% virulence reduction when compared to wild-type R19 (Figure 1B).

### 3.2. Determination of T-DNA Integration Pattern

**Figure 2:**
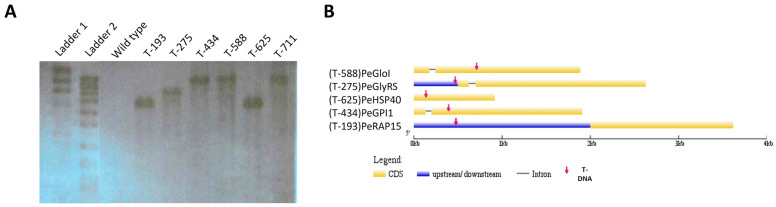
DNA gel-blotting analysis of T-DNA. (**A**) WT (R19) genomic DNA was digested with restriction endonucleases *PAC I*. Molecular markers are indicated on the left. (**B**) Diagram of *P. expansum* genes interrupted by T-DNA insertion. A total of five transformants were used for localizing T-DNA insertion through thermal asymmetric interlaced polymerase chain reaction (TAIL-PCR). Five genes were found potentially irrupted by T-DNA insertion in coding sequences (CDS) or untranslated regions (UTR), including GloI (glyoxalases I), GlyRS (glycyl-tRNA synthetase), HSP40 (heat-shock protein 40), GPI1 (N-acetylglucosaminyl transferases), and RAP15 (U8 small nucleolar RNA-associated protein 15). The gene structure figure was generated with Gene Structure Display Server (v2.0; http://gsds.cbi.pku.edu.cn/index.php accessed on 2 February 2019).

Using a probe derived from the T-DNA, DNA-blotting with the genomic DNA of six selected transformants (T-193, T-275, T-434, T-588, T-625, T-711) was performed. As shown in Figure 2, no band was observed in the lane carrying the DNA of wild-type *P. expansum* (R19), while one unique band appeared on each lane of the transformants, clearly showing a single insertion of the T-DNA in the genome of examined transformants. Except for T-588 and T-711 (which were inserted into the same locus), other transformant bands have different sizes, suggesting the positional diversity of the T-DNA insertion in those transformants within the *P. expansum* R19 genome (Figure 2).

### 3.3. Identification of Loci Interrupted by T-DNA Insertion

Thermal asymmetric interlaced polymerase chain reaction (TAIL-PCR) was implemented on selected virulence mutants to isolate DNA sequences flanking the T-DNA insertion. Using the combination of three T-DNA-specific primers and four different degenerate primers, LB and RB flanking sequences were obtained from transformants T-193 (PEXP_001700), T-434 (PEXP_051540), T-588 (PEXP_003290), T-625 (PEXP_008080), and T-711 (PEXP_003290), while only the LB flanking sequence was identified from T-275 (PEXP_016530). TAIL-PCR was not able to amply the RB of T-DNA in T275, indicating a possible excision failure of the right border during the integration of T-DNA into the R19 genome. DNA sequencing revealed flanking sequences of a border of T-DNA were exclusive for each transformant, indicating the uniqueness of the T-DNA integration (Table S3). Interestingly, T-588 and T-711 were found to share identical RB and LB flanking sequences, suggesting they are derived from the same event of T-DNA integration. We thus did not pursue T-711 for further study. Nevertheless, these flanking sequences allowed us to localize the T-DNA integration sites and identify putative integration-affected genes by comparing the adjacent genome sequence of the T-DNA inserting locus in transformants and *P. expansum* R19 (Figure 2B, Table 1).

For mutant T-193, a putative ORF located downstream 1518 bp to the insertion locus was found to be the coding sequence closest to the T-DNA. Blasting against the NCBI protein database revealed that the putative protein exhibited the highest percent identity (99.82%) to a U8 small nucleolar RNA-associated protein 15 (RAP15) in *Penicillium viridicatum*. The putative protein has the UTP15_C motif conserved in other RAP15 members, and is thus named PeRAP15. For T-275, T-DNA was found to be inserted 33 bp upstream of a putative start codon of an ORF. The putative protein encoded by the ORF showed the highest similarity (87.3%) to the glycyl-tRNA synthetase (GlyRS) in *Penicillium digitatum* according to the BLAST results against the NCBI protein database. No conserved motif was identified for this protein sequence (GlyRS) or from other species that also contained the putative protein. This ORF was thus named PeGlyRS. For T434, T-DNA was inserted into the coding area of a putative gene. Its amino acid sequence displayed high similarity to N-acetylglucosaminyl transferases (GPI1). Alignment of this sequence to other GPI1s showed the sequence contained the conserved domain. Consequently, this putative gene was named PeGPI1. For T588, T-DNA was found to be in the coding sequence of a putative ORF. Blasting against the NCBI database showed that the putative protein had the highest identity to glyoxalases (GloIs) from other species. Like other GloIs, this protein also contained a conserved VOC motif responsible for the isomerization of the spontaneously formed hemithioacetal adduct between GSH and 2-oxoaldehydes (such as methylglyoxal) into S-2-hydroxyacylglutathione [25]. For T-625, T-DNA was inserted into a putative ORF that codes a protein showing high similarity to the heat-shock protein 40 (HSP40) cochaperone Jid1 in *P. digitatum*. The protein, together with other HSP40s, shared a common DNA-J domain, a typical motif of a chaperone [26]. This mutant, T-625, was investigated and characterized by [16], and shown to be involved in secretion processes, toxin production, and virulence in apple fruits.

### 3.4. Expression Profiles for Genes up- and Downstream of the T-DNA Insertion

**Figure 3:**
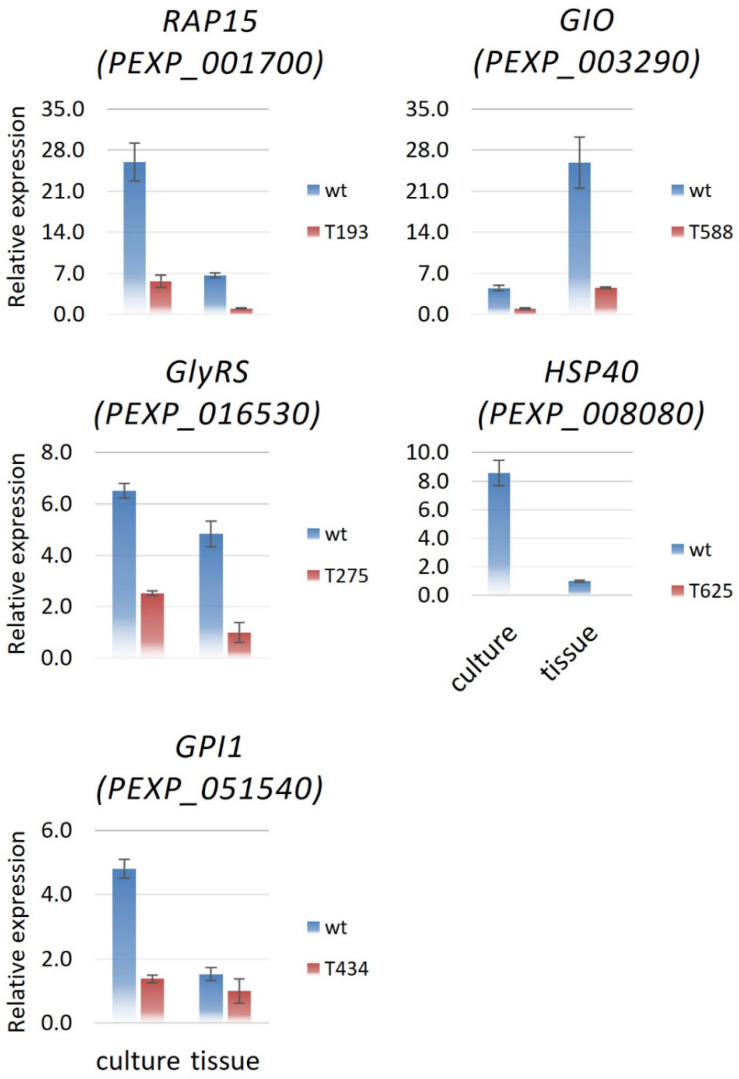
Expression patterns of genes potentially responsible for the virulence mutation of transformants in PDA culture and decayed apple tissues. Total RNA was extracted from PDA culture and decayed apple tissues after 10 days post inoculation (DPI). The data represent the mean value (±SD) of three replicates.

To assess how the T-DNA insertion affects the transcription of candidate genes, we investigated the expression of candidate genes in both wild-type (R19) and mutant fungi from liquid-medium culture and *P. expansum*-decayed apple fruit tissue at 10 DPI. All candidate genes, including RAP15, GloI, GlyRS, HSP40, and GPI1, are transcribed in fungi from both in vitro culture and decayed apple tissue, indicating they are active in vivo (Figure 3). Among the five genes, RAP15, GlyRS, HSP40, and GPI1 displayed significantly lower expression in the decayed apple tissue than in the culture medium, while GloI showed five-times-higher expression in decayed tissue than in the culture medium. In the culture at 10 DPI, transcription of all candidate genes was heavily reduced (>62%) in transformants. In decayed tissues at 10 DPI, transcription of four genes (RAP15, GloI, GlyRS, and HSP40) was significantly lower (>79%) in transformants, and only the expression of GPI1 was decreased when comparing apple and media culture conditions. This observation suggests T-DNA insertion in both noncoding and coding areas can disrupt the transcription of these genes. Considering that the T-DNA insertion occurred in the coding region in three transformants, including T434, three genes, including GPI1, were expected to have aberrant-sized transcripts, or may not be transcribed due to the T-DNA insertion. Hence, these directly impacted loci serve as functional null mutants.

### 3.5. Targeted Single-Gene Deletion or Knockdown Strain Phenotypes

**Figure 4:**
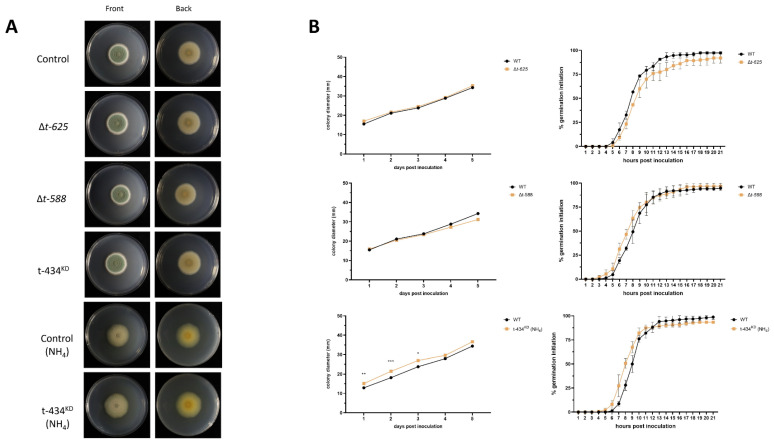
Targeted mutant morphology and physiology. (**A**) Mutant appearance after cultivation in GMM plates for 7 days using initial inoculum of 10^6^ spores at 25 °C. Knockdown mutant was assessed in GMM and GMM-amended media (using NH_4_ as a nitrogen source). (**B**) Radial growth measurements 7 days post inoculation in GMM, and germination initiation assay on liquid minimal media (LMM) 21 h post inoculation at 25 °C. * *p* < 0.05, ** *p* < 0.01, *** *p* < 0.001.

To confirm that the observed T-DNA phenotypes are due to the interruption of the identified genes, we aimed to generate deletion strains for all the identified loci. We successfully obtained deletion mutants for the genes that had T-DNA insertions T-625 and T-588. Since obtaining deletion strains for the genes that contained T-DNA insertions in T-193, T-262, or T-434 was not possible, we sought to obtain knockdown strains. We successfully generated a knockdown strain for T-434, but not for T-193 or T-262. The genotypes for the obtained strains are listed in Table S2. Thus, we hypothesize that the deletion or silencing of these loci results in a lethal phenotype. After obtaining the deletion and knockdown strains, we assessed the radial growth and germination initiation rates of these when compared to the control strain, and found no statistically significant differences between our deletion mutants and the control strain (Figure 4A,B). We observed that, when grown on ammonia as a nitrogen source, t-434*^KD^* exhibits a slight but statistically significant increase in radial growth when compared to the control strain in the same media. Upon assessing the germination initiation rate for t-434*^KD^* using ammonia as the nitrogen source, we did not observe any significant difference between the control and knockdown strains (Figure 4A,B).

### 3.6. ∆T-588 Strain Shows Reduced Lesion Decay Diameter In Vivo

**Figure 5:**
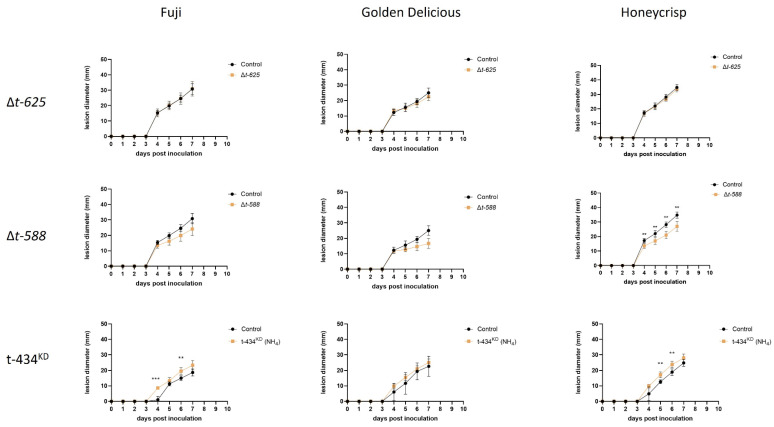
Apple inoculation assays for targeted mutant strains. Apple lesions were measured for 7 days in Fuji, Golden Delicious, and Honeycrisp cultivars. ** *p* < 0.01, *** *p* < 0.001.

**Figure 6:**
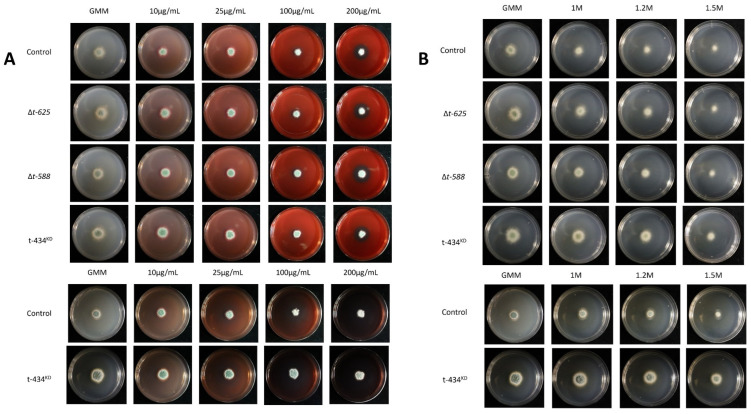
Congo Red and sorbitol susceptibility plate assay. The mutants were assessed at different concentrations of GMM amended with (**A**) Congo Red or (**B**) sorbitol. GMM using ammonium salts was used to evaluate t-434*^KD^*. Images were obtained 5 days post inoculation.

After generating the deletion and knockdown mutants, these strains were assessed for variation in lesion development in ‘Fuji’, ‘Golden Delicious’, and ‘Honeycrisp’ apples. The primary objective was to test the hypothesis that these genes were involved in virulence during apple fruit decay, as evidenced by the initial screening performed using the T-DNA mutants. Interestingly, neither ∆*t-625* nor t-434*^KD^* showed reduced lesion diameter when compared to the control strain on any of the tested apple cultivars (Figure 5). As for ∆t-588, it was observed that there was reduced lesion diameter in ‘Honeycrisp’ apples when compared to the control strain. The same trend holds for ∆t-588 in both ‘Fuji’ and ‘Golden Delicious’, although the differences for these cultivars are not statistically significant. Unexpectedly, we observed that t-434*^KD^* showed a slight but significant increase in lesion diameter in ‘Fuji’ and ‘Honeycrisp’ cultivars when compared to the control strain (Figure 5). In addition, we subjected these mutants to cell wall and membrane stressors, Congo Red and sorbitol, in different concentrations. None of the strains showed increased or reduced susceptibility to the assessed stressors (Figure 6).

### 3.7. ∆t-588 Shows Reduced Colony Diameter When Exposed to Methylglyoxal

**Figure 7:**
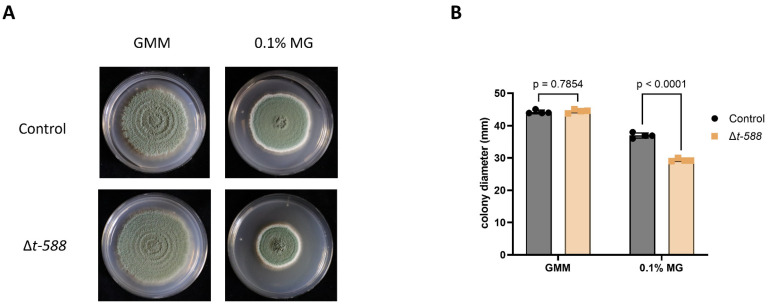
Methylglyoxal (MG) plate assay. Deletion strain Δ*t-588* was assessed for in vitro susceptibility of 0.1% MG in GMM-amended plates using an initial inoculum of 100 spores. (**A**) Plates were imaged and the (**B**) colony diameters quantified 12 days post inoculation.

While bioinformatically assessing possible gene function for the identified loci, it was noted that the locus interrupted in the ∆*t*-588 mutant contained a glyoxalase domain. Therefore, it was hypothesized that PEXP_003290 could function in detoxifying methylglyoxal. To test this, ∆t-588 and the control strain were cultured in GMM and GMM supplemented with methylglyoxal at a final concentration of 0.1% [27]. After incubating the plates for 14 days, it was observed that the ∆t-588 colony had decreased radial growth when compared to the control strain (Figure 7).

## 4. Discussion

To develop novel management strategies for blue mold decay, one needs to understand the molecular underpinnings and biochemical pathways that modulate virulence in *P. expansum*. In this study, a forward and reverse genetics approach was used to discover and characterize new genes important in *P. expansum* virulence during apple fruit decay. After screening 448 random T-DNA insertion mutants for reduced lesion diameter in apples, we obtained 6 mutants (T-191, T-434, T-262, T-588, T625, and T-711) and identified 5 loci that are involved in *P. expansum* virulence. Upon identification of the disrupted genes, we aimed to obtain deletion mutants for each of the loci and were successful in obtaining two deletion mutants (∆*t-625* and ∆*t-588*) and one knockdown strain (t-434*^KD^*). In general, we found similar technical trends, patterns, and limitations with the AMT system as in other studies [9,28]. For example, a common technical challenge in AMT is its low efficiency and left-border excision failures, which was encountered in this study [28]. In addition, deletion or knockdown strains were unable to be generated for some of the identified loci, which is a common occurrence that has been observed in other fungal pathogens, like *Magnaporthe oryzae*, *Fusarium oxysporum*, and *Botrytis cinerea*.

### 4.1. UTP15 (T-DNA Mutant T-193)

The U3 small nucleolar RNA-associated protein 15 (UTP15) is involved in the nucleolar processing of pre-18S rRNA. This protein is typically found together with the other two WD proteins, i.e., CIRH1A and WDR43, and are found in the nuclear matrix fraction of HeLa cells. They are contained in the t-UTP subcomplex of SSU processomes, and have been shown to bind directly with each other and to bind tightly to the FC, independent of rRNA transcription, and are involved in ribosome biogenesis [29]. Interestingly, in a forward genetic screening in zebrafish, a mutation in *utp15* could affect vascular patterning in a p53-dependent manner in zebrafish embryos [30]. In another study, UTP15 was shown to be upregulated when *A. nidulans* was treated with abscisic acid (ABA) [31]. Aside from the discovery of this locus in other fungal systems, our work is key in nature regarding this finding in *Penicillium* spp.

### 4.2. Glycyl-tRNA Synthetase (T-DNA Mutant T-275)

Glycyl-tRNA synthetase (GlyRS) can charge tRNAs with glycine, and thus plays an important role in protein synthesis, which makes this enzyme ubiquitous in all cell types [32]. It has been reported that GlyRS interacts with elongation factor 1-delta (EEF1D), which participates in the enzymatic delivery of aminoacyl tRNAs to the ribosome as a subunit of the elongation factor-1 complex [33]. GlyRS has been shown to exert diverse functions, like acting as gene regulators, involvement in intron splicing, and angiogenesis, in addition to their role in translation [34]. Conserved signature indels (CSIs) were identified in GlyRS protein sequences across Xanthomonadales and a limited number of Beta- or Gamma-proteobacteria that mostly act as plant or human pathogens, indicating the potential involvement of GlyRS in pathogenesis [35]. Translative efforts have been directed at the development of new antibiotics, which would inhibit the activity of aminoacyl-tRNA synthetases, including GlyRS from pathogens such as *Enterococcus faecalis*, *Staphylococcus aureus*, *Streptococcus pneumoniae*, *Helicobacter pylori*, *Mycobacterium tuberculosis*, or *Candida albicans* [36,37]. Recently, the characterization and function of GlyRS proteins in plant-specific fungal pathogens have not been reported. Our finding indicates the potential involvement of the GlyRS protein in the virulence process during apple decay in *P. expansum.* Systematic functional analysis of GlyRS protein genes could shed additional light on the rationale of this class of proteins in the fungal virulence of *P. expansum* and other fungal pathogens with varying biology and lifestyle.

### 4.3. Glyoxalase (T-DNA Mutants T588 and T711, and ∆t-588 Mutant)

Glyoxalase I (GloI) is part of the glyoxalase system that catalyzes the detoxification of the endogenous reactive metabolite, methylglyoxal (MG), which is produced as a byproduct of glycolysis [38]. GloI is responsible for the isomerization of the hemithioacetal formed spontaneously from α-oxoaldehyde and GSH to S-2-hydroxyacylglutathione derivatives RCOCH(OH)-SG^®^ RCH(OH)CO-SG in the cytosol of cells [39]. This reaction decreases the steady-state concentrations of the metabolic byproduct methylglyoxal and other reactive acyclic α-oxoaldehydes that mediate glycation reactions [40,41]. Glycation is a nonenzymatic glycosylation that impairs the functioning of biomolecules by bonding sugar molecules to a protein or lipid molecule. This process is implicated in many age-related chronic diseases, such as cardiovascular and Alzheimer’s disease [41,42,43,44]. The primary function of glyoxalase I is the detoxification of α-oxoaldehydes as part of the enzymatic defense against glycation. In addition, glyoxalases have been implicated in bacterial and fungal virulence. Glyoxalase A (*gloA*) was found to have a role in the virulence activation of *Listeria monocytogenes*, and similarly to ∆*t-588*, *gloA* mutants were sensitive to exogenous MG [45]. In *C. albicans*, the deletion of *glx3* results in an avirulent phenotype along with decreased biofilm filamentation [46]. However, the role of glyoxalase in *Penicillium*–apple fruit interactions has not previously been ascertained. It is envisioned that the discovery of this new locus, encoding a glyoxalase enzyme, is important for the fungus to tolerate the generation of toxic byproducts from the central metabolism (e.g., methylglyoxal) that are generated during decay. Hence, this finding adds a new, logical perspective to the concept of fungal virulence factors, in that it is critical for the pathogen to detoxify metabolites from the primary metabolism to enable sufficient self-protection and to ensure optimal lesion/decay during the apple fruit colonization.

### 4.4. GPI1 (T-DNA Mutant T-434 and t-434^KD^)

Glycosylphosphatidylinositol (GPI) is a glycolipid that acts as an anchor to many cell-surface proteins, and in fungi, can also be found in their cell walls [47,48]. GPI anchors are bound to the C-terminus of proteins through post-translational modifications [48]. GPI-anchored proteins have diverse roles in cell biology and include cell–cell interactions, mammalian or protozoan antigens, and enzymes, among others [47]. Synthesis of GPI and its transfer to proteins comprise a series of 23 steps that take place in the endoplasmic reticulum (ER) [49]. In mammals, the first GPI synthesis step is catalyzed by a protein complex of six core subunits that include PIGQ or its homolog in *Saccaromyces cerevisiae* GPI1 [49]. In *S. cerevisiae*, *gpi1*-deficient strains show a temperature-sensitive phenotype, are unable to synthesize GPI in vitro, and, when cultured in permissive temperatures, have a separation defect after budding [50]. Due to the importance of GPI, a previous study proposed using GPI anchor biosynthesis as a potential target for developing antifungals [51]. This idea was explored through the development of host-induced gene silencing (HIGS) against many plant-pathogen fungal species and was evidenced by a patent [52]. While we could not generate a knockout strain, temperature was not considered as a selection criterion for screening transformants in this study. Hence, it cannot be ruled out that the possibility of obtaining a complete null mutant using temperature and other parameters that center around GPI biology and biochemistry. The assays within this study’s evaluation knockdown strain suggests that the level of GPI1 is sufficient to foster GPI biosynthesis, as no impact was observed in the growth nor virulence defect, in contrast to its T-DNA mutant counterpart. This is the first time a GPI-biosynthesis-related gene has been studied in *Penicillium* spp., and these findings shed light on additional aspects of protein post-translational modifications and promising antifungal targets as new areas for decay control.

### 4.5. HSP40 (T-DNA Mutant T-625 and ∆t-625)

Hsp40 proteins are divided into three classes, I, II, and III, which differ in their structural patterns [53,54]. The major role of Hsp40s are to regulate Hsp70’s activity through an ATP-dependent interaction [55,56,57]. Hsp70 and Hsp40 are involved in protein folding, transportation, and localization, and thus act in many cellular activities, including fugal–plant interactions [55,56,57,58]. For instance, Hsp70 in *Colletotrichum gloeosporioides* displayed higher expression in the infected coffee plant [59]. Hsp70 proteins of *M. oryzae* were also found to accumulate in the endoplasmic reticulum (ER) of fungi and were responsible for disease development in rice plants [60]. These proteins were observed during the cell-wall degradation of host plants, and thereby assisted virulence when a plant pathogenic fungus, *Rhizoctonia solani*, was attacking soybean seedlings [61].

Besides acting as an ATP-dependent chaperone, Hsp40 has also been found to independently assist viruses to invade host cells. In mammalian cells, double-stranded RNA-dependent protein kinase (PKR) is a key regulator of the antiviral innate immune response [62]. In *P. expansum*, Hsp40 exhibited lower expression (about 0.12-fold) in infected apple tissue than in culture at 10 dpi (Figure 3). This unexpected observation might be caused by the delay in examination regarding the transcriptional level of Hsp40. We only examined the expression of Hsp40 with highly decayed tissue at 10 dpi in this study. However, a previous study showed that the possible roles of this gene involve modulating protein secretion through the ER and vesicle-mediated transport [16].

### 4.6. Loci That Were Recalcitrant to Gene Deletion/Functional Analysis

Deletion mutants for the genes disrupted in T-DNA mutants T-191 and T-262 could not be obtained during this study. It can be hypothesized that these genes may be essential due to both their bioinformatic prediction and their recalcitrant behavior upon transformation with both deletion and knockdown constructs. Previous studies have also documented a lack of success in generating targeted deletion mutants after a random mutagenesis approach. For example, in another study, a random ATM was performed in *B. cinerea*, and the authors could not generate targeted deletion mutants for one out of three characterized genes [9]. In addition, two of these genes are involved in RNA processing, which is an essential cellular process; so, even though there may be redundancy, we hypothesize that removing any of these components can be lethal to the organism. One of the benefits of ATM is the possibility of studying essential genes due to the random insertion, which can provide a T-DNA mutant knockdown strain, as evidenced in this study.

While it is unfortunate that other loci did not phenocopy the T-DNA mutants and/or resulted in loci that are presumed lethal, this information provides abundant opportunities for further investigation. Mechanisms behind why the mutants did not phenocopy remain elusive and may be due to a variety of factors. These may include the transcriptional, translational, and post-translational impacts of the locus and/or the surrounding loci from the T-DNA insertion [9]. Perhaps applying comparative genomic and/or transcriptomic approaches to the strains to determine pleiotropic or off-target effects of the T-DNA insertion may be warranted. Previous studies in yeast, where the entire KO library was sequenced, showed compensatory mechanisms were activated upon deletion of a single locus [63]. Perhaps this may be a parallel situation in our system that warrants a more detailed omics-based investigation. Our approach to utilizing the genes with presumed lethal phenotypes can be further explored using dsRNA interference in single and combinations to not only assess gene function but also to determine if these loci can be targeted using dsRNAs as a form of control strategy which has been implemented in other cropping systems [64,65].

## 5. Conclusions

In general, this study provides new insights into the complex biological picture of *P. expansum*–apple fruit interactions and the genes that are involved in the decay process. As shown by the diversity of loci discovered from this study, the virulence and the decay process represent a complex, well-orchestrated system that has multiple overlapping systems involving secretion, membrane components, RNA processing, protein synthesis, and metabolite detoxification. For the first time, a random T-DNA mutant library was generated, and identified five loci important to virulence in this species. Although some of these loci seem to be essential, identification through this method was possible. Two deletion mutants and one knockdown strain were generated that were evaluated for virulence. Locus PEXP_003290 was found to phenocopy the T-DNA mutant and supports the hypothesis that this locus acts as a glyoxalase, which is important for full virulence during apple fruit decay. The generation of this random T-DNA transformant library also serves as a resource for investigating other aspects of *P. expansum* biology. Future studies should focus on obtaining a more comprehensive library by increasing the total number of transformants. It can then be utilized to investigate other aspects of *P. expansum* biology, like differential production of specialized metabolites, tolerance or sensitivity to various fungicides, the metabolism of substrates at cold temperatures, spore germination defects, protein secretion, and natural product synthesis. Any of these avenues that explore the functional genetic basis of a given biological process regulating the decay process are envisioned to set the foundation for future blue mold decay strategies.

## Figures and Tables

**Table 1:** T-DNA mutant list and annotation description.

T-DNA Mutant Name	Locus ID	Annotation Description
T-193	PeRAP15 (PEXP_001700)	U3 small nucleolar RNA-associated protein 15, C-terminal
T-275	PeGlyRs (PEXP_016530)	hypothetical protein
**T-588/T-711**	PeGIO (PEXP_003290)	hypothetical protein/VOC containing domain/serine rich protein
**T-434**	PeGPI1 (PEXP_051540)	phosphatidylinositol N-acetylglucosaminyltransferase subunit Q/GPI1
**T-625**	Blistering1 (PEXP_008080)	DNA-J-domain-containing protein

## Data Availability

Data is contained within the article and supplementary material.

## Supplementary Materials

The following supporting information can be downloaded at: https://www.mdpi.com/article/10.3390/jof9111066/s1, Table S1: List of primers for generating deletion and knock down mutants; Table S2: List of Strains used in this study; Figure S1: T-DNA of pPK2 hph; Figure S2: Southern Blot confirmation for deletion and knock-down strains and design schematics. Table S3: List of BLAST results for loci interrupted by T-DNA insertion.
