# Supplementary material for: Mining the Penicillium expansum Genome for Virulence Genes: A Functional-Based Approach to Discover Novel Loci Mediating Blue Mold Decay of Apple Fruit
Source: J Fungi (Basel). 2023 Nov 1;9(11):1066. doi: 10.3390/jof9111066 (PMC10672711; doi:10.3390/jof9111066)
Supplement: Supplementary file 1 [file jof-09-01066-s001.zip › jof-2658416-supplementary.pdf]

Table S1- List of primers for generating deletion and knockdown mutants.

Primer Name	Primer Sequence
DL_Pexp_Blistr_5'_F	AAA GGG GGA AGA AGT GAA AGG CG
DL_Pexp_Blstr_5SF	ACA AGA GTT CGA TTC AAT ATG CAG AGG C
DL_Pexp_Blstr_5_NF	AAT GCG ATC ACC GTA TTC GTT GCG
DL_Pexp_Blstr_5_R	CTC TAT TGA CCT ATA GGA CCT GAG TGA TGC TGT CAC ATG GTT TCT CTT CAG ACA GC
DL_Pexp_Blstr_3_F	AAG TTG AGC ATA ATA TGG TCC ATC TAG TGC TTC TGT TAG ATG AGG ATA TGA TTG CAT GG
DL_Pexp_Blistr_3N_R	ACC AAT GAC GAT GTT CGT CAC AAT CC
DL_Pexp_Blistr_3_R	AAC AGA TGC CAA GGT TAG AAT ATG AGG
DL_Pexp_Blistr_3S_R	TAC TAT GGA CAT CCC CCA TTC TCG
DL_PEXP_003290_3N_R	ACG TCT ACA AGA CCG ACC GAC C
DL_PEXP_003290_3_F	ATG GCC AGA GTA TGC GGC AAG TCA TGA TGA TGG TGG TGA TGT TCT TTT GCA TCT TAT TTC
DL_PEXP_003290_5N_F	GCA CTT CTG CCC ATC GTA TAT TTG G
DL_PEXP_003290_5_F	GTT CTA GGG AAT CTG GGA ATA CTG G
DL_PEXP_003290_5_R	CAC AGT GGA GGA CAT ACC CGT AAT TTT CTG TGC GAA GGT TTG AAG TCG ACG TGC
DL_PEXP_003290_3_R	GGT TGA TAT CAA TGA GGG GAT CAT GG
DL_PEXP_003290_ORF_F	ATT TCT GGA TGA CCG AGA CTA AGC C
DL_PEXP_003290_ORF_R	TGC CGT CGT AGA TTG TGC TGG C
DL_PEXP_T-434-KD_3_F	TAT ATT ATT TTC CTA TCC CAT ACT CTC ACA ATG CAC CCC TTT TCC GTC CTC AC
DL_PEXP_T-434_KD_3N_R	CAA GGC TAG CGA AAT CGG GTC G
DL_PEXP_T-434_KD_3_R	ATA TCA ACC TGT TGG GCT GTT GCC G
DL_Pexp_AfumiPyrG_F	CAG AAA ATT ACG GGT ATG TCC TCC AC
DL_Pexp_AfumiPyrG_R	TCA TGA CTT GCC GCA TAC TCT GG
DL_Pexp_niia_promoter_F	TAT ATG GCC AGA GTA TGC GGC AAG TCA TGA GAT GGC GGG CGC GGT GAT T
DL_niiA_niaD_promoter_R	TGT GAG AGT ATG GGA TAG GAA AAT AAT ATA GAG

Table S2-List of Strains used in this study.

Strain Name	Genotype	Strain Source
TDL 9.1	Δ*ku70*::*six* Δ*pyrG::six-**β**-rec-hph-six*	Wang et al., 2021
TWW 12.1	*Δku70::six ΔpyrG::six*	Wang et al., 2021
TWW 13.1 (Control)	Δ*ku70*::six Δ*pyrG*, pyrG::*Afumi* pyrG in ku70 locus (excised hygromycin cassette)	Wang et al., 2021
TDL 15.1.2.3.4	Δ*ku70*::*six* Δ*pyrG*::*six-**β**-rec-hph-six* Δ*blistering1*::*Afumi pyrG*	This study
TDL 22.1.2.5	Δ*ku70*::six Δ*pyrG*::*six* Δ*pexp_003290*::*A.fumipyrG* (T-588)	This study
TDL 37.1	Δ*ku70*::*six* Δ*pyrG*::*six* pyrG::niaDp::*pexp_051540* (T-434 -knock down)	This study

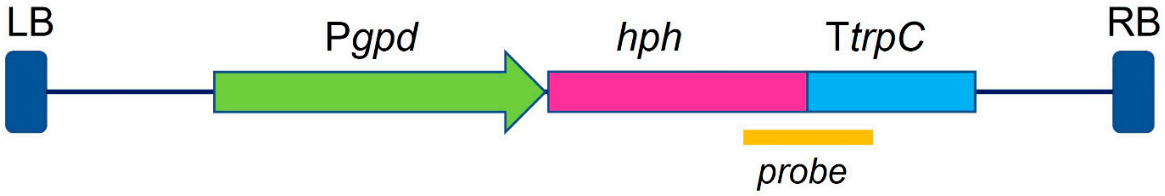

Figure S1- T-DNA of pPK2 *hph*. The T-DNA cassette contains the following components: LB, left border; PgpdA, *Aspergillus nidulans* glyceraldehyde-3-phosphate dehydrogenase promoter; hph, hygromycin B resistance gene; TtrpC, A. nidulans trpC terminator; RB, right border.

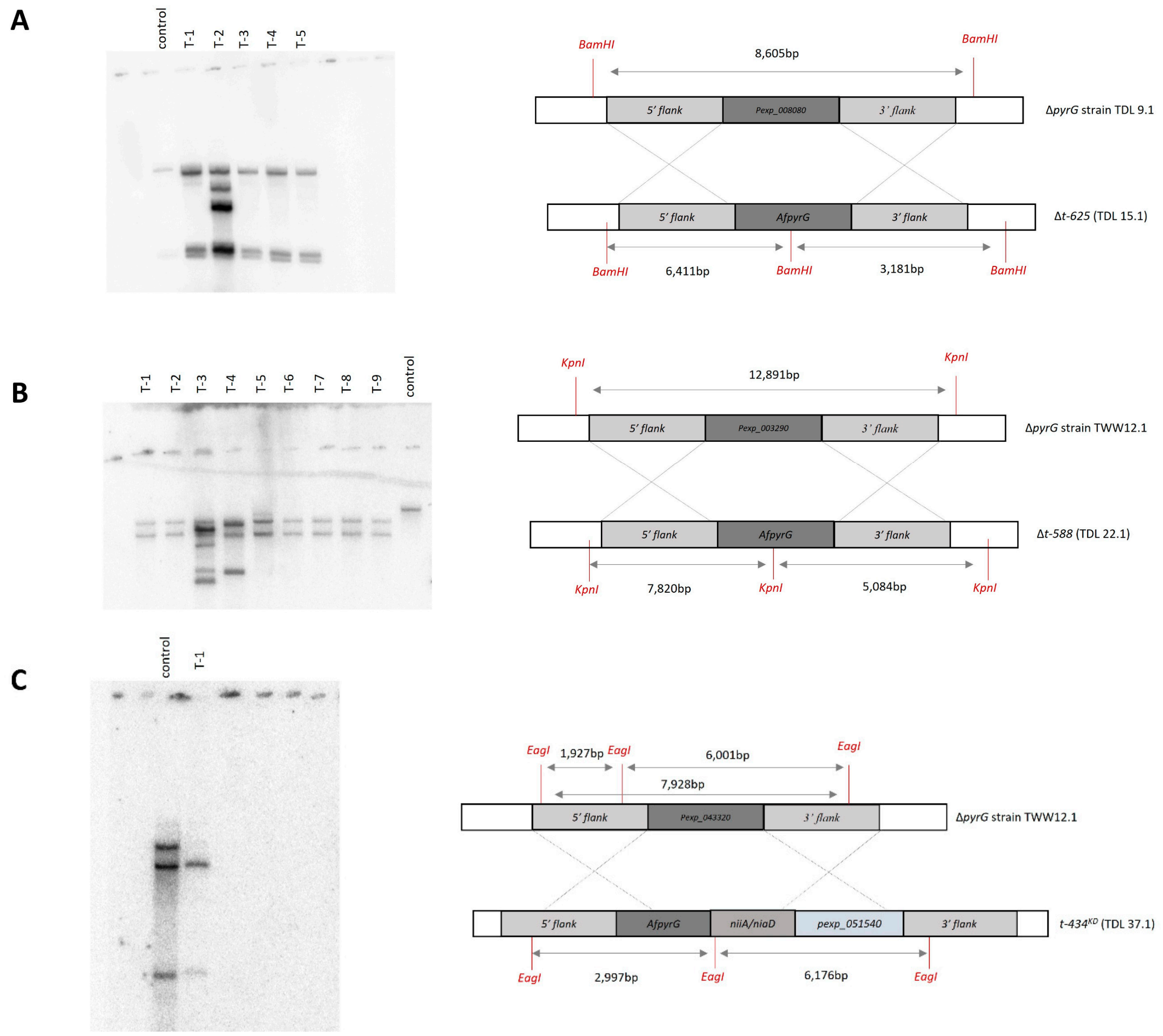

Figure S2- Southern Blot confirmation for deletion and knock down strains and design schematics. A) Δ*t-625* B) Δ*t-588 C) t-434*^{KD}. The Southern blots were designed to obtain a distinct band patterned between the WT and mutant strains when using the 5' and 3' flanks simultaneously as probes.

Table S3- List of BLAST results for loci interrupted by T-DNA insertion.

#	Transformant	Integration Location	Left Border	% identity/Reference ID	Right Border	% identity/Reference ID
1	T-89	5'	*P. chrysogenum* Wisconsin 54-1255 complete genome	(69%) AM920428.1	*P. expansum* Chaperone, tailless complex polypeptide 1	(69%) XM_016745867.1
2	T-91	n.d.	n.d	n.d.	*P. expansum* Chaperone, tailless complex polypeptide 2	(83%) XM_016745867.1
3	T-163	5'	Excision failure	T-DNA cloning vector	*P. expansum* Chaperone, tailless complex polypeptide 1	(78%) XM_016745867.1
4	T-193	5'	*P. chrysogenum* Wisconsin 54-1255 hypothetical protein	(100%) XM_002563011.1	*P. chrysogenum* Wisconsin 54-1255 complete genome	(86%) AM920435.1
5	T-272	n.d.	Excision failure	T-DNA cloning vector	*P. chrysogenum* Wisconsin 54-1255 complete genome	(77%) AM920428.1
6	T-275	3'	*P. expansum* hypothetical protein	(100%) XM_016747523.1	Excision Failure	T-DNA cloning vector
7	T-381	n.d.	Excision failure	T-DNA cloning vector	*P. chrysogenum* Wisconsin 54-1255 complete genome	(73%) AM920436.1
8	T-404	n.d.	Excision failure	T-DNA cloning vector	n.d.	n.d.
9	T-413	n.d.	Excision failure	T-DNA cloning vector	*P. expansum* Chaperone, tailless complex polypeptide 1	(78%) XM_016745867.1
10	T-434	n.d.	*P. expansum* N-acetylglucosaminyl transferase	(94%) XM_016745122.1	*P. expansum* DNA mismatch repair protein	(95%) XM_016745121.1
11	T-489	5'	Excision failure	T-DNA cloning vector	*P. expansum* Chaperone, tailless complex polypeptide 1	(78%) XM_016745867.1
12	T-588	3'	*P. expansum* hypothetical protein	(92%) XM_016739231.1	*P. chrysogenum* Wisconsin 54-1255 complete genome	(70%) AM920427.1
13	T-625	coding region	*P. expansum* Heat shock protein	(100%) XM_016738171.1	*P. expansum* Heat shock protein	(74%) XM_016738171.1

14	T-711	3'	*P. expansum* hypothetical protein	(96%) XM_016739231.1	*P. chrysogenum* Wisconsin 54-1255 complete genome	(80%) AM920427.1
